# Improving health professionals’ capacity to respond to the climate crisis in Africa: outcomes of the Africa climate and health responder course

**DOI:** 10.3389/fpubh.2025.1617723

**Published:** 2025-10-15

**Authors:** Danielly de P. Magalhães, Cecilia Sorensen, Nicola Hamacher, Haley Campbell, Hannah N. W. Weinstein, Patrick O. Owili, Alex R. Ario, Glory M. E. Nja, Charles A. Michael, Yewande Alimi, Hervé Hien, Woldekidan Amde, Sokhna Thiam, Vincent Pagiwa, Shawn M. D’Andrea, Caroline M. Gichuki, Marian Offei, Joanes Atela, Sean M. Patrick, Bruce Struminger, Margaret Kaseje

**Affiliations:** ^1^Global Consortium on Climate and Health Education, Mailman School of Public Health, Columbia University, New York, NY, United States; ^2^Department of Emergency Medicine, Columbia University Irving Medical Center, Columbia University, New York, NY, United States; ^3^Research and Related Capacity Strengthening Division, African Population and Health Research Center, Nairobi, Kenya; ^4^International Association of National Public Health Institutes (IANPHI), Africa Network, Addis Ababa, Ethiopia; ^5^Uganda National Institute of Public Health, Kampala, Uganda; ^6^Association of Schools of Public Health in Africa (ASPHA), Nairobi, Kenya; ^7^School of Allied Health Sciences, Kampala International University, Ishaka, Uganda; ^8^Health Services Management and Health Policy Unit, Department of Public Health, University of Calabar, Calabar, Nigeria; ^9^Africa Centres for Disease Control and Prevention, Addis Ababa, Ethiopia; ^10^School of Public Health, University of the Western Cape, Cape Town, South Africa; ^11^West Africa Regional Office, African Population and Health Research Center, Dakar, Senegal; ^12^Okavango Research Institute, University of Botswana, Maun, Botswana; ^13^Brigham and Women's Hospital, Boston, MA, United States; ^14^Institute for Human Development, Aga Khan University, Nairobi, Kenya; ^15^Africa Research and Impact Network (ARIN), Nairobi, Kenya; ^16^School of Health Systems and Public Health, University of Pretoria, Pretoria, South Africa; ^17^Division of Infectious Diseases, University of New Mexico Health Sciences Center, Albuquerque, NM, United States; ^18^The ECHO Institute, University of New Mexico Health Sciences Center, Albuquerque, NM, United States

**Keywords:** climate adaptation, climate change education, Africa, capacity building, health professionals

## Abstract

**Introduction:**

The fragile health systems in Africa worsen climate-related health impacts, making capacity building essential to strengthen adaptation and resilience. The Africa Climate and Health Responders Course was developed to address the urgent need for climate and health education among African health professionals. Organized by the Global Consortium on Climate and Health Education (GCCHE) in collaboration with ASPHA, Africa CDC, WHO AFRO, Project ECHO, and other regional partners, the course aimed to enhance awareness, communication skills, and preparedness in responding to climate-related health challenges.

**Methods:**

Delivered online via Zoom with over 11 sessions (September 17–October 22, 2024), the course featured expert lectures, case studies, and live discussions. Simultaneous interpretation in English, French, and Portuguese ensured broad accessibility. Participants who attended at least 70% of live sessions and passed the final exam received a certificate. A longitudinal survey was applied to understand the course impact.

**Results:**

The course attracted 7,572 registrants, with 89% from Africa. While 3,500 participants attended at least one session, only 1,657 participated (1,607 from Africa) attended 70% or more of the sessions and completed the final survey. Participants held positions in government (31%), Non-Governmental Organizations (NGOs) (27%), academia (24%), private sector (11%), and others (7%). Their main professional backgrounds were public health (33.2%), medicine (16.3%), and environmental health (13.2%). The majority of participants (66%, *n* = 1,100) had never received prior training in climate and health; among them, 36% (*n* = 392) were students and 64% (*n* = 708) were not students.

**Discussion:**

The course significantly improved participants’ self-reported confidence and perceived preparedness, with increases in: climate-health awareness (+22%); confidence in risk communication (+40%); preparedness for adaptation and resilience (+36-37%), and professional responsibility in climate-health action (+21%). These findings highlight not only the feasibility and effectiveness of virtual training in this context, but also the opportunity for scaling such initiatives to build a climate-resilient health workforce across Africa. Skilled professionals are key to fostering multi-stakeholder collaboration, integrating climate-health education, and engaging communities—efforts that require sustained investment in capacity building to institutionalize competencies and strengthen public health systems and policies over the long term.

## 1 Introduction

Climate change is widely recognized as one of the most significant global health threats of the 21st century, with profound implications for morbidity, mortality, and health systems worldwide (1). The African continent, despite contributing minimally to global greenhouse gas emissions, is one of the most vulnerable and least prepared regions to cope with climate-related health impacts. To build climate-resilient health systems in Africa, there is an urgent need to equip health professionals with the knowledge and skills to understand, communicate, and respond to climate-related health threats.

While nearly 20% of the global population lives in Africa, the World Meteorological Organization (WMO) estimates it produces less than 10% of global emissions (2). At the same time, Africa bears a disproportionate burden of risks related to public health, agriculture, migration, conflict, and economic and infrastructural development in the face of climate change (3). Extreme weather events such as droughts, floods, and cyclones have intensified in frequency and severity, exacerbating existing health inequities across the region.

In September 2023, Mediterranean Cyclone Storm Daniel provided a glimpse into the continent’s future, resulting in widespread destructive flooding, 11,000 confirmed casualties, and approximately 20,000 people affected (4). Additionally, 2023 was one of the hottest years in Africa and was the warmest year on record in many countries, according to 124 years of historical temperature data (3). Droughts, driven by extreme temperatures, continue to exacerbate food insecurity and adversely affect vulnerable populations’ health in Africa. In Ethiopia alone, nearly 16 million people are food insecure due to drought, floods, desert locusts, COVID-19, conflict, and economic shocks (5). In the Horn of Africa, over 30 million people faced drought-related food insecurity between 2020 and 2022 (6).

Climatic change is also escalating the intensity of vector-borne diseases such as malaria, dengue, and Rift Valley fever, driven by shifting ecological conditions and resulting in prolonged breeding seasons for mosquitoes and other vectors (7). Africa bears a disproportionate share of the global malaria burden, accounting for 94% of malaria cases and 95% of malaria deaths worldwide (8). Additionally, food and water insecurity, resulting from prolonged droughts and unpredictable rainfall patterns, has heightened the risk of malnutrition, waterborne diseases, and other health complications, particularly affecting vulnerable populations such as children and pregnant women (9). In 2024, the combined effects of El Niño and climate change led to unprecedented drought conditions in Southern Africa. The region experienced the driest February in over a century (10), resulting in devastating impacts on agriculture and food security, endangering millions of lives across countries such as Angola, Botswana, the Democratic Republic of Congo, Malawi, Mozambique, Namibia, Zambia, and Zimbabwe (11).

The impact of climate change on public health is significantly dependent on the preparedness of health systems, whose quality and timeliness of preparedness and response are contingent upon the capacity of health professionals to identify, prevent, prepare for, and respond to climate-related health impacts. Capacity building enables effective climate action by equipping people and institutions to adapt, mitigate emissions, and build resilience. The Intergovernmental Panel on Climate Change (IPCC) states that “With proactive, timely and effective adaptation, many risks for human health and well-being could be reduced and some potentially avoided (very high confidence)” (12). The health impacts of climate change in Africa are exacerbated by fragile health systems characterized by inadequate infrastructure, workforce shortages, and limited financing (13). To overcome existing and future threats to health and health systems, comprehensive training for health professionals on climate-related health threats is essential to build climate adaptation and thereby improve service delivery during acute climate events and build climate-resilient health systems (14).

To address this urgent need, the Climate and Health Responders Course, a global series of free, evidence-based and certificate-granting courses led by the Global Consortium on Climate and Health Education (GCCHE) at Columbia University, was developed and implemented for the African continent. Specifically, the goal of this course was to: (1) increase health professionals’ communication about climate and health, (2) equip health professionals with knowledge and skills that could be readily incorporated into practice, and (3) engage health professionals with climate and health initiatives within their communities. The program also emphasizes the role of health professionals as trusted voices in their communities, advocating for policies and interventions that protect public health in the face of climate change.

The aims of this study were:

1. To evaluate the effectiveness of the course in meeting its stated goals.
2. To assess whether participation in a 15-h live-virtual course increased the capacity of health professionals to address climate and health threats.
3. to provide insights for scaling similar educational interventions in other regions facing significant climate-related health threats and provide a framework on which to build more comprehensive climate change and health education in Africa.
4. To lay the foundation for long-term systemic impact by fostering leadership, collaboration, and advocacy skills that can contribute to institutional change, including policy development, health system resilience, and sustainability.

For that, we conducted a longitudinal study of course participants, to assess whether the 15-h live-virtual course increased health professional capacity to address climate and health threats. By providing an evidence-based curriculum tailored to the specific climate threats faced by African communities, the course was designed to equip participants with the tools necessary for effective climate adaptation, mitigation, and resilience-building efforts. This manuscript details the methodology and outcomes of this course and outlines its effectiveness in improving climate and health capacity among African health professionals. It aims.

## 2 Materials and methods

### 2.1 Partnership

The Climate and Health Responders Course, a region-based initiative of the GCCHE, equips health professionals with fundamental knowledge on the intersections of climate change and health. To ensure the course effectively addressed Africa’s unique challenges and to ground the course in the realities of African health systems, foster local ownership, and ensure the inclusion of region-specific expertise and case studies, it was developed and implemented collaboratively among a working group with representatives from key African organizations. These included the Association of Schools of Public Health in Africa (ASPHA), Africa Centers for Disease Control and Prevention (Africa CDC), the World Health Organization Regional Office for Africa (WHO AFRO), the International Association of National Public Health Institutes (IANPHI) Africa Network, Project ECHO, the Climate & Health Africa Network for Collaboration and Engagement (CHANCE Network), the Africa Research and Impact Network (ARIN), and the African Population and Health Research Center (APHRC). Representatives from these institutions met weekly from April to June 2024 to adapt the standard Climate and Health Responder Course curriculum and to identify regional experts to deliver lectures and case studies. From July to August, 2024, efforts focused on inviting speakers and developing a marketing strategy to maximize participation and impact.

### 2.2 Program structure

This course was designed for health professionals across varying levels of experience and different specialty and subspecialty backgrounds. The curricular foundation of this educational initiative was the GCCHE core competencies for health professionals, a set of highly-vetted global educational standards which cover climate and health analytic skills and knowledge, communication and collaboration, policy, and public health and clinical practice competencies (15), which were systematically translated into progressive learning objectives using Bloom’s taxonomy. This hierarchical framework guided our curriculum design by classifying educational objectives from basic knowledge acquisition to complex analytical skills, while ensuring the objectives were applicable to all health professional backgrounds. The session topics and learning objectives were reviewed by the local partners to ensure they accurately reflect African climate challenges, socio-economic determinants of health, cultural knowledge, and local needs.

The course was delivered online twice a week via the Zoom webinar platform and consisted of 11 sessions, each lasting 90 min (see Appendix 1 for the course curriculum). The course was implemented from September 17th to October 22nd, 2024. Each session featured a 45-min lecture followed by a 20-min case study presentation(s) and a discussion segment during which participants could engage directly with speakers utilizing a ‘question and answer’ discussion format.

The lectures and case studies were primarily presented by African subject matter experts and practitioners representing public, private and academic entities from throughout Africa, thus offering a comprehensive and regionally relevant overview. These speakers also served as examples of professionals currently working within the climate and health field within the geographic region thereby providing participants with a base for further engagement with the field.

This case-based learning, which integrates theoretical knowledge with real-world scenarios, fosters critical thinking and problem-solving abilities by immersing participants in practical situations that reflect regional challenges they may face in climate and health projects, effectively bridging the gap between theory and practice. Synchronous online discussion is central to problem-based learning, promoting constructive reflection and analysis.

To enable wide accessibility and inclusivity, the course was primarily conducted in English, complemented by simultaneous interpretation in French and Portuguese. The course was offered free of charge.

### 2.3 Intended audience and recruitment

This educational initiative aimed to engage physicians, nurses, allied health professionals, national or local public health workers, hospital administrators, health system leaders, health educators, policymakers, environmental health professionals (trained experts who work to prevent disease and promote health by identifying and addressing environmental risks that impact human well-being) and government officials.

Participants were invited through outreach conducted via the GCCHE network and partner networks, using email and social media to reach both individuals and professional groups. Engagement was further supported by a WhatsApp group for registrants that provided reminders, shared materials, and facilitated direct interaction with course coordinators once participants were registered.

### 2.4 Registration and longitudinal survey

All participants who registered for the course were invited to enroll in the longitudinal study. Study protocol was approved and classified as exempt by Columbia University Institutional Review Board (AAAR4912). A pre-course survey was included in the registration process, which featured demographic questions and seven longitudinal survey questions. The longitudinal survey was modified from prior GCCHE course surveys and developed by GCCHE experts in climate change and health with the intent to align with the course’s learning objectives and assess participants’ climate awareness, confidence, and behaviors related to their ability to communicate the health impacts of climate change, their preparedness to act on climate and health plans, and their sense of responsibility in contributing to climate adaptation and mitigation efforts within their communities (See Appendix 2 for survey questionnaire).

The survey responses were measured using a Likert scale, with options ranging from 1 to 10. For a question measuring *impact*, a response of 1 indicated “Not relevant—Climate change does not impact my professional practice,” while a 10 indicated “To a large extent—Climate change impacts all facets of my professional practice.” For questions assessing *confidence*, a response of 1 indicated “Not confident,” while a response of 10 indicated “Very confident.” For questions evaluating preparedness, a response of 1 indicated “I feel unprepared,” while a 10 indicated “I feel very prepared.” For questions assessing participants’ sense of professional *responsibility*, a response of 1 indicated “I feel no responsibility,” while a 10 indicated “I feel a very high sense of responsibility.”

After completing the final exam, participants were asked to respond to a post-course longitudinal survey, which repeated the pre-course questions to assess any changes in their perception of knowledge, communication confidence, preparedness, and sense of professional responsibility related to climate change.

### 2.5 Course participation and certification

Participants who attended 7 or more of the live Zoom sessions and passed the final exam with a score of 70% or higher at the end of the course were eligible for a Certificate of Participation. Attendance was recorded using Zoom attendance reports. The day after the course concluded, all participants received an invitation via email and WhatsApp with a link to complete the final exam on Qualtrics. In order to test the knowledge gained, participants were required to complete the final exam in one sitting within a five-day period following the final course session.

The final exam was specifically designed to assess participants’ understanding of the science-based content covered in each session topic, aligning closely with the course learning objectives, slides, and key topics discussed throughout the sessions. It consisted of 25 multiple-choice and true/false questions, each worth 1 point, resulting in a maximum possible score of 25. The exam was administered only once, at the end of the course, without pre- or post-testing, which limits the possibility of conducting comparative or item-level analyses over time. There was no time limit to finish the exam after initiation. The final exam was offered in English, French and Portuguese. Upon successful completion participants received a Certificate of Participation, issued on behalf of the collaborating partner organizations.

### 2.6 Analysis

All data from registration, course participation and both pre- and post-surveys was analyzed using R software (Version 2024.09.1 + 394). Additional analysis of statistical significance using Wilcoxon signed rank tests and two-sample proportion tests was completed using the same software. The analysis included only participants who completed both the pre- and post-course longitudinal survey. Data was first analyzed and checked for duplicate entries by participants. Out of the duplicate entries, the participants’ most recent submission was kept for data analysis.

For each longitudinal survey question, the change in the number of participants and the percent change from the pre-survey to the post-survey were calculated for each answer choice. This change was determined by subtracting the number of participants’ responses in the post-survey from those in the pre-survey. The resulting difference represents the shift in participant responses over time and represents the impact of the course on participant responses. Statistical significance levels (*p*-levels) comparing initial survey responses to post-survey responses were determined using a two-sample proportion test. For individual questions, significance was assessed through a Wilcoxon signed-rank test, a non-parametric method suitable for paired data comparisons. Finally, the end-of-course exam scores were assessed by examining the median scores for all participants as well as the 25th and 75th percentiles to assess the distribution of participant performance.

## 3 Results

### 3.1 Demographics and participation

**Figure 1:**
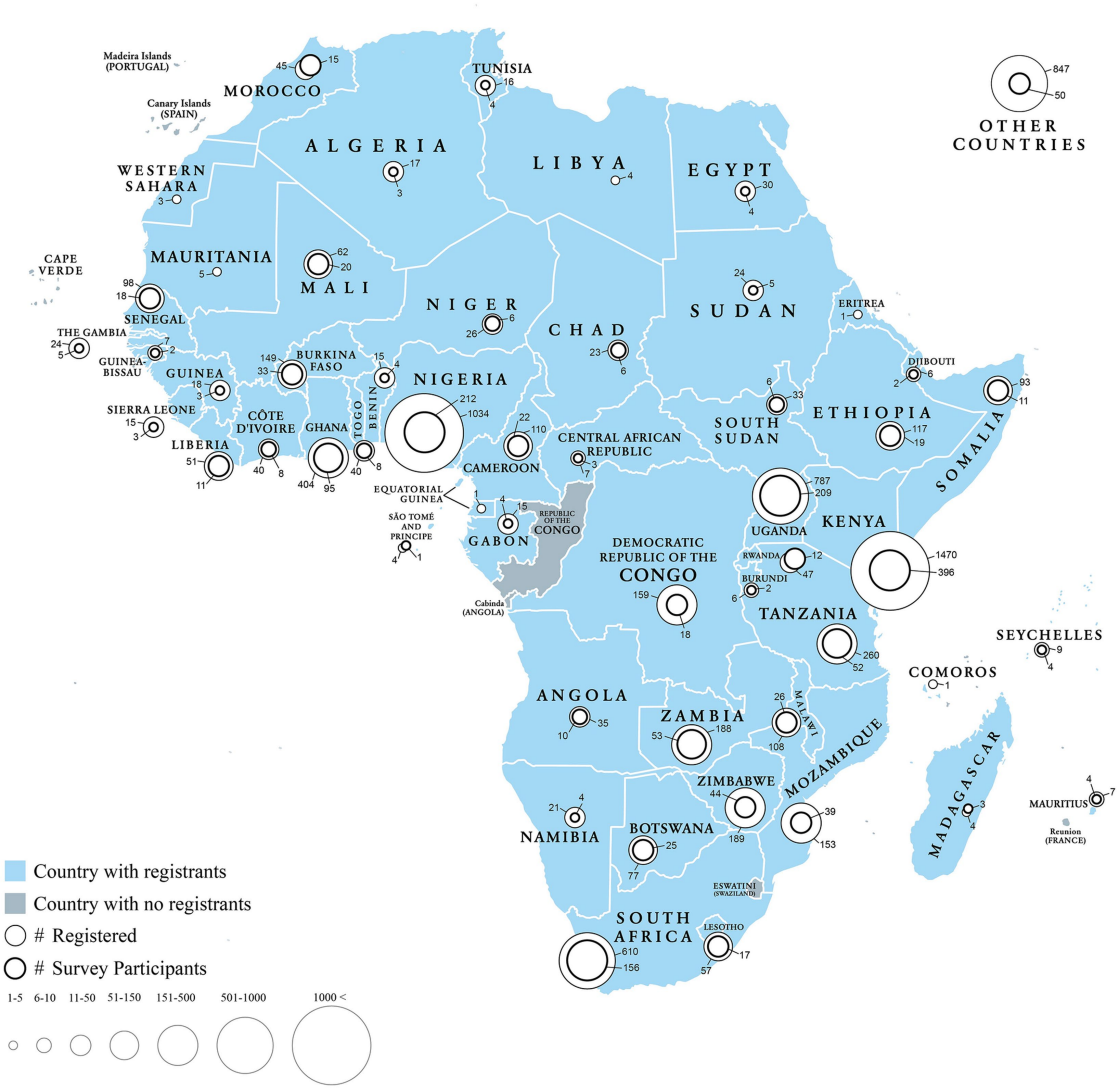
Distribution of course registrants and final survey participants across African countries (*n* = 1,607).

Of the 7,572 individuals who registered for the course, 6,725 (89%) were from Africa (Figure 1; Appendix 3 for complete list of countries) representing 52 out of 54 African countries, over 35 different health-related fields and a variety of general places of work. Approximately 31% of registrants came from governmental offices, 27% from civil-society organizations, 24% from academic institutions, 11% from private organizations, and 7% from other organizations (Table 1).

**Table 1:** Location of work of course registrants and survey participants.

Place of work	Registered (*n* = 7,572)	Survey participants (*n* = 1,657)
Academic, research institution	1780 (23.51%)	407 (24.56%)
Government, inter-governmental	2,380 (31.43%)	510 (30.78%)
Non-governmental organization, non-profit	2087 (27.56%)	442 (26.67%)
Other	497 (6.56%)	124 (7.48%)
Private sector	828 (10.94%)	174 (10.5%)

Table 2 presents the reported occupation of course registrants and survey participants. Of 7,572 course registrants, the majority were in the field of public health (*n* = 2,515, 33.2%), followed by medicine (*n* = 1,238, 16.3%) and environmental health (*n* = 1,000, 13.2%). Among survey participants (*n* = 1,657) public health (*n* = 566, 34.2%), medicine (*n* = 265, 16%) and environmental health (*n* = 247, 14.9%) were the highest represented occupations.

**Table 2:** Reported occupation of course registrants and survey participants.

Reported occupation	Registered (*n* = 7,572)	Survey participants (*n* = 1,657)
Advanced Practice Provider	5 (0.1%)	NA
Biostatistics	108 (1.4%)	22 (1.3%)
Clinical Social Worker	5 (0.1%)	3 (0.2%)
Communications/Marketing	41 (0.5%)	10 (0.6%)
Consultant	65 (0.9%)	13 (0.8%)
Dentist	11 (0.1%)	6 (0.4%)
Doctor	68 (0.9%)	16 (1%)
Economics/Finance	91 (1.2%)	16 (1%)
Educator	150 (2%)	24 (1.4%)
Emergency Responder	24 (0.3%)	3 (0.2%)
Engineering	47 (0.6%)	15 (0.9%)
Environmental Engineering	63 (0.8%)	11 (0.7%)
Environmental Health	1,000 (13.2%)	247 (14.9%)
Epidemiology	407 (5.4%)	76 (4.6%)
Health Administration	39 (0.5%)	7 (0.4%)
Information Technology	52 (0.7%)	10 (0.6%)
Journalism/Media	25 (0.3%)	7 (0.4%)
Laboratory Technician	114 (1.5%)	33 (2%)
Law	37 (0.5%)	6 (0.4%)
Medicine	1,238 (16.3%)	265 (16%)
Mental Health	48 (0.6%)	7 (0.4%)
Natural/Physical Science	141 (1.9%)	41 (2.5%)
Nurse Infirmier	229 (3%)	45 (2.7%)
Occupational Health	30 (0.4%)	5 (0.3%)
Other	271 (3.6%)	55 (3.3%)
Pharmacist	143 (1.9%)	21 (1.3%)
Philanthropy	5 (0.1%)	NA
Physical/Occupational/Speech Therapy	29 (0.4%)	5 (0.3%)
Public Health	2,515 (33.2%)	566 (34.2%)
Public Health Informatics	191 (2.5%)	37 (2.2%)
Public Health Policy	132 (1.7%)	23 (1.4%)
Public Policy	42 (0.6%)	10 (0.6%)
Social Work	88 (1.2%)	21 (1.3%)
Urban Planning/Architecture	20 (0.3%)	5 (0.3%)
Veterinarian	98 (1.3%)	26 (1.6%)

Among registrants, 1,657 individuals completed the longitudinal survey, representing 47 African countries (Figure 1). Among survey participants, 61% (*n* = 1,015) were in professional practice, while 39% (*n* = 642) were students. The majority of respondents (66%, *n* = 1,100) had never received prior training in climate and health, whereas 34% (*n* = 557) had some form of prior training in this area. Among those who had never received training, 36% (*n* = 392) were students and 64% (*n* = 708) were professionals. Among the 708 practicing professionals, 35% (*n* = 247) worked in government, and 18% (*n* = 125) were professionals in academia, 31% (*n* = 220) were from non-governmental organizations, and 12% (*n* = 87) were from the private sector. Among the participants who had received prior training (*n* = 557), 45% (*n* = 250) were students, while 55% (*n* = 307) were practicing professionals. Among the 307 professionals, 33% (*n* = 102) were from the government, and 18% (*n* = 55) were professionals working in academia, 31% (*n* = 96) were from non-governmental organizations, and 12% (*n* = 37) were from the private sector.

### 3.2 Longitudinal survey

**Figure 2:**
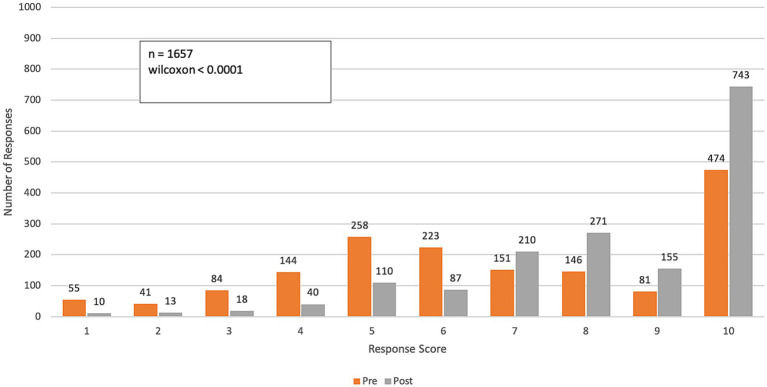
Pre- and post-course distribution of responses to *Q1: “To what extent do the impacts of climate change on health affect the work you do in your professional practice?”* Responses were rated on a range from 1 (“Not relevant—Climate change does not impact my professional practice”) to 10 (“To a large extent—Climate change impacts all facets of my professional practice”).

**Figure 3:**
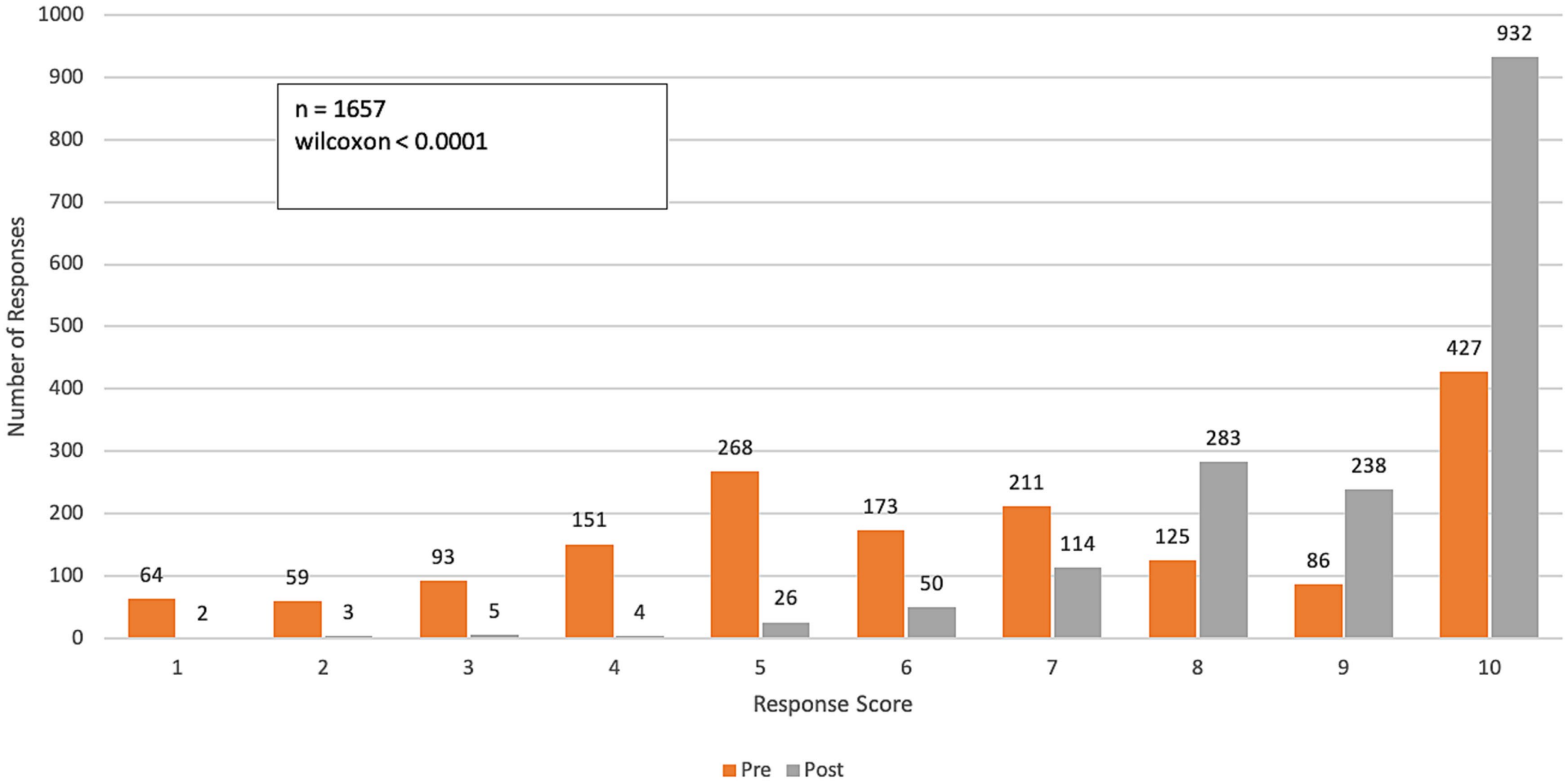
Pre- and post-course distribution of response scores to Q2: “*How confident do you feel in communicating with work colleagues about the health impacts of climate change?*” Responses were rated on a range from 1 (“not confident”) to 10 (“very confident”).

**Figure 4:**
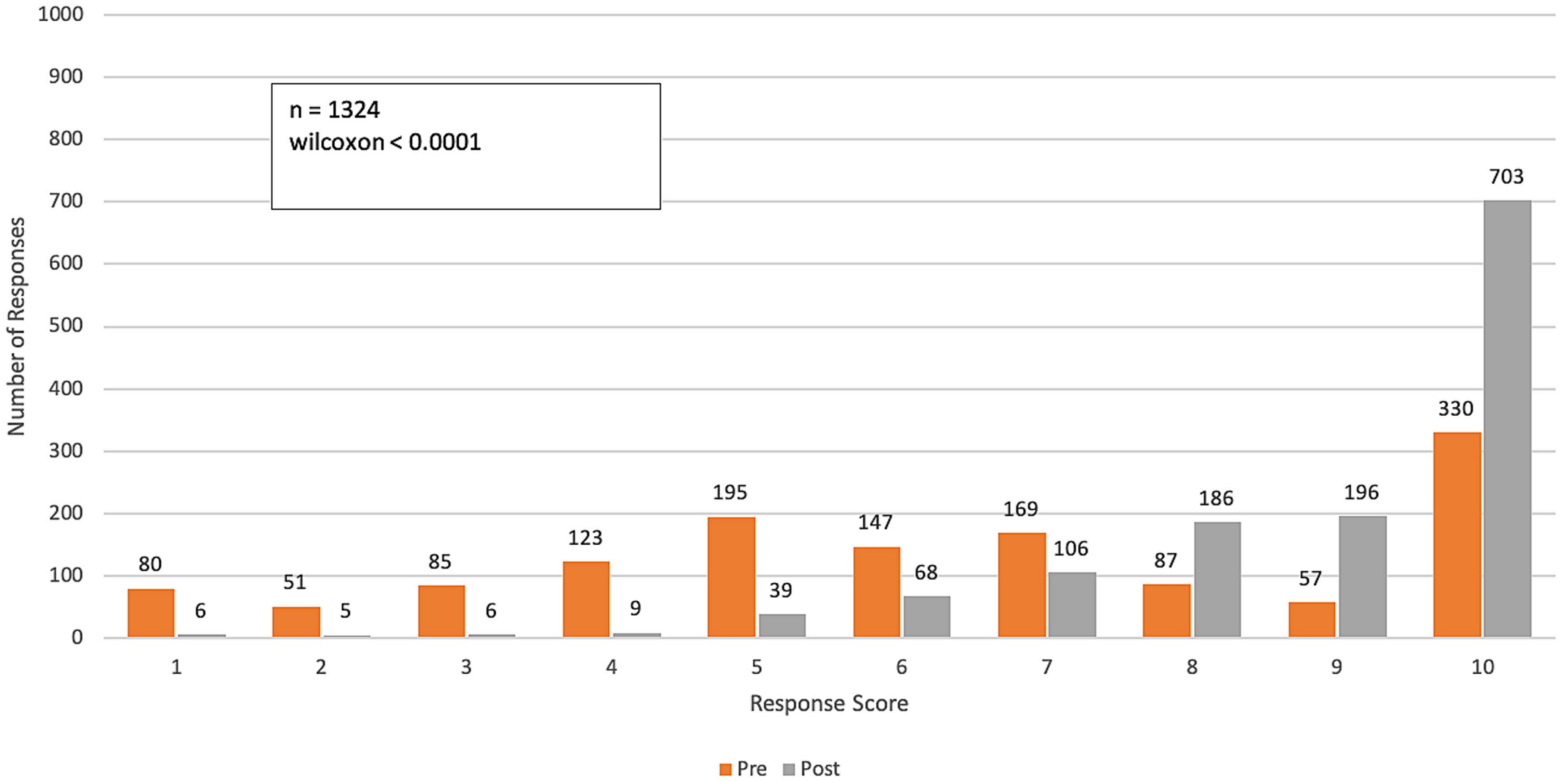
Pre- and post-course distribution of response scores to Q3: *How confident do you feel communicating with PATIENTS about the health impacts of climate change?* Responses were rated on a range from 1 (“not confident”) to 10 (“very confident”).

**Figure 5:**
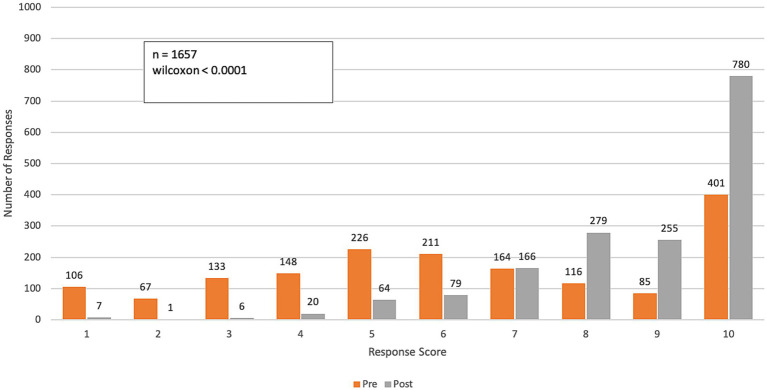
Pre- and post-course distribution of response scores to Q4: *“To what degree do you feel prepared to: Help your community adapt to the health threats of climate change?”* Responses were rated on a range from 1 (“unprepared”) to 10 (“very prepared”).

**Figure 6:**
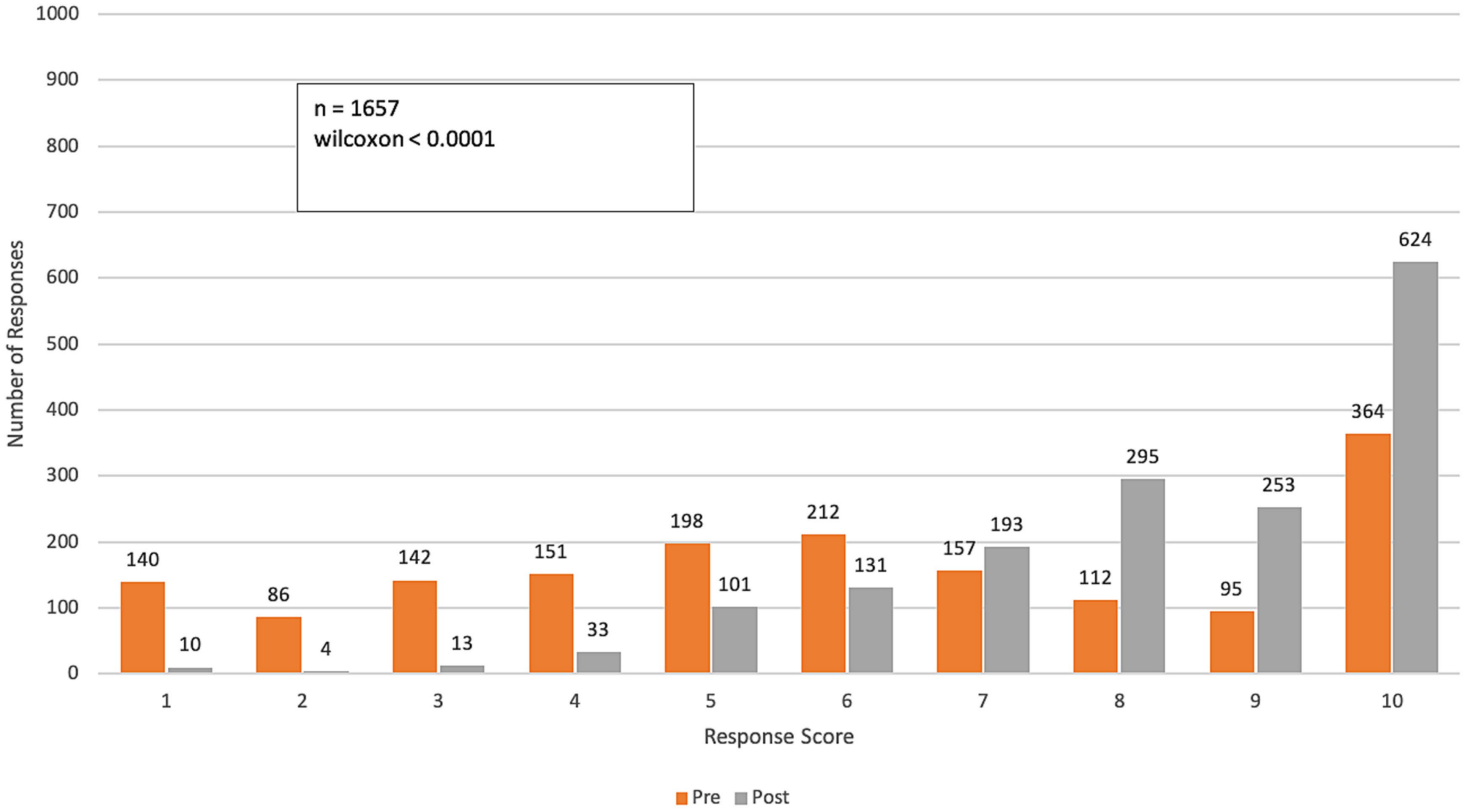
Pre- and post-course distribution of response scores. *Q5 asked, “To what degree do you feel prepared to: Help your health system decarbonize and become more resilient to the impacts of climate change?.”* Responses were rated on a range from 1 (“unprepared”) to 10 (“very prepared”).

**Figure 7:**
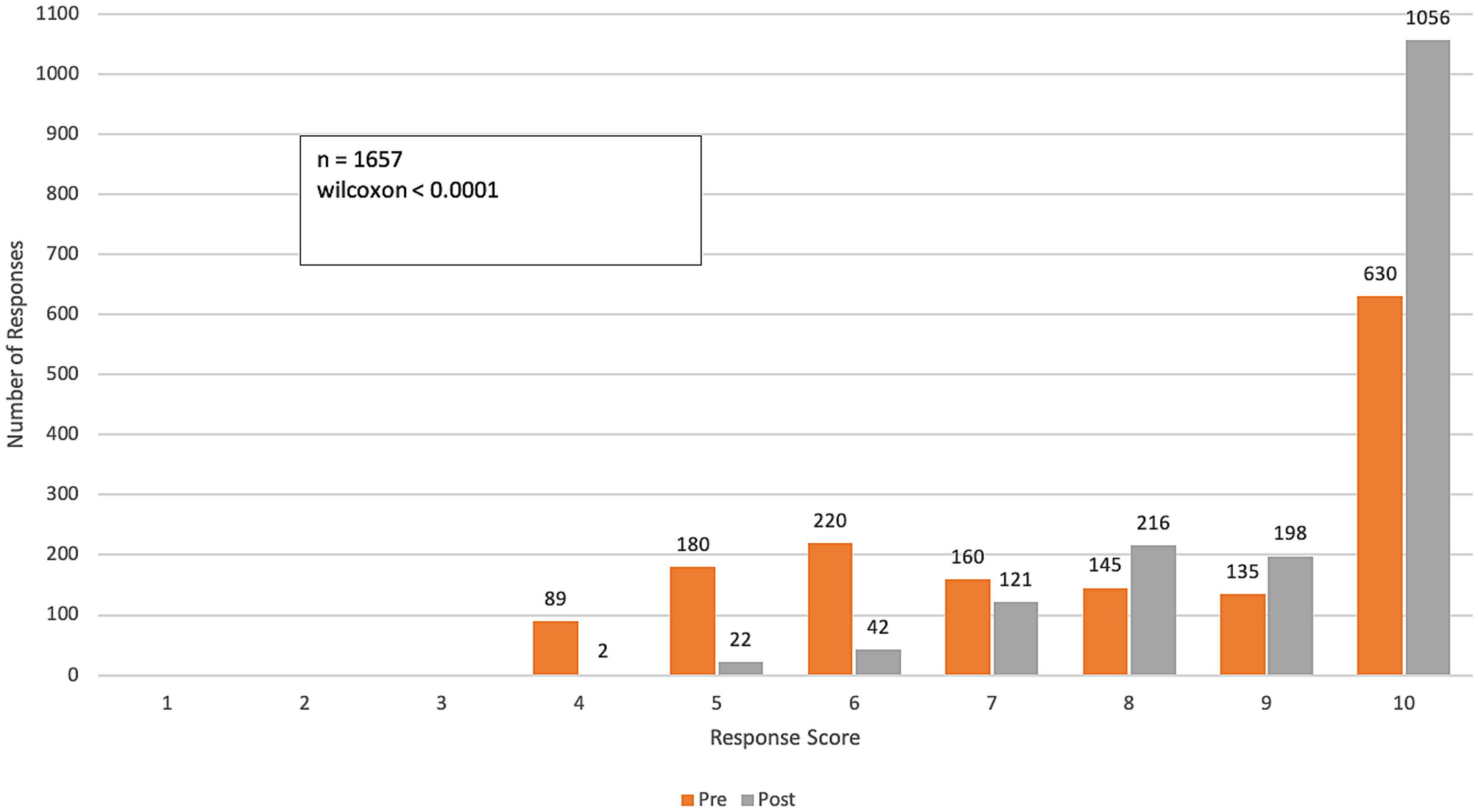
Pre- and post-course distribution of response scores to Q6: “*To what extent do you feel a professional responsibility to help your community adapt to the health threats of climate change?”* Responses were rated on a Likert scale from 1 (“no responsibility”) to 10 (“very high responsibility”).

**Figure 8:**
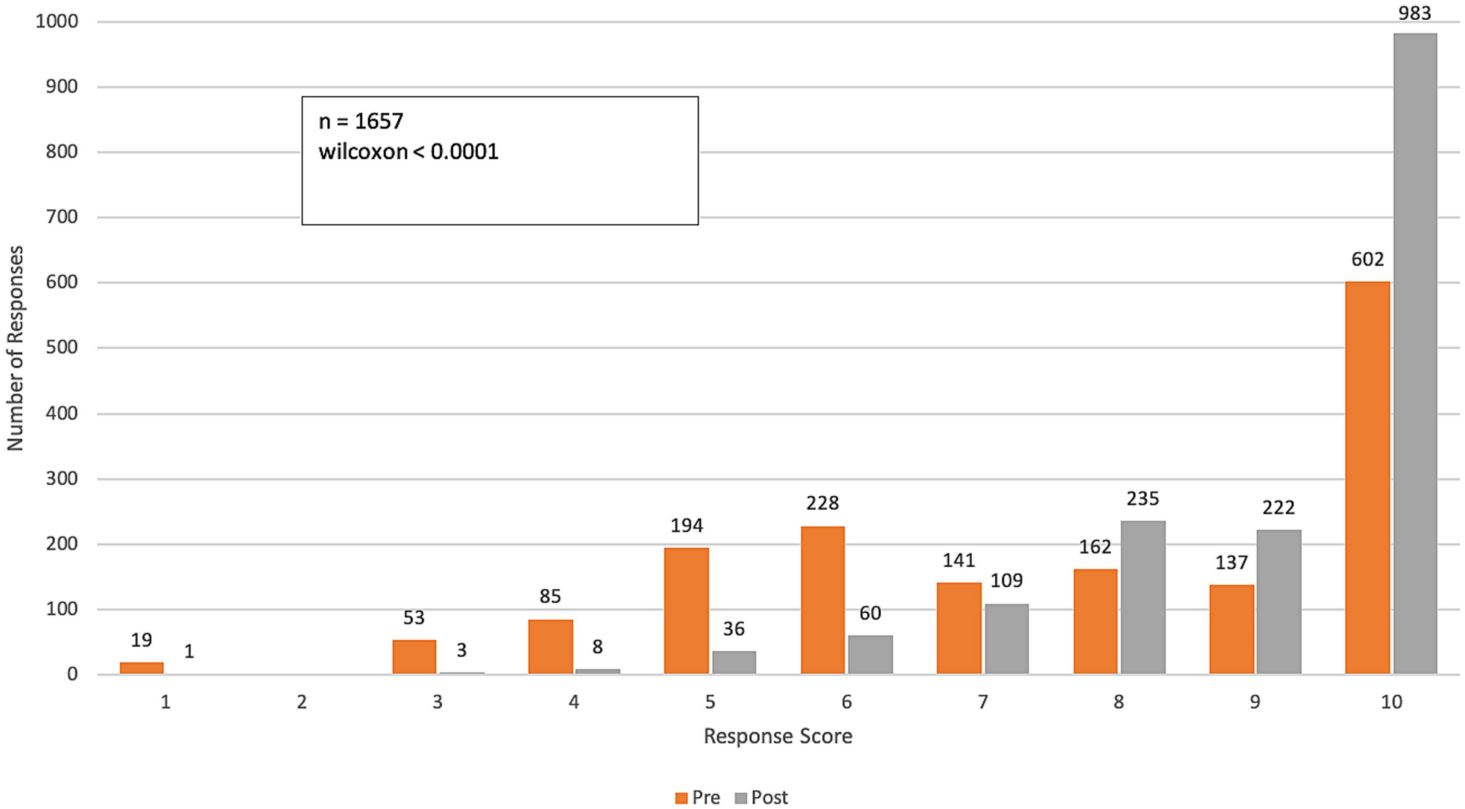
Pre- and post-course distribution of response scores to Q7: *“To what extent do you feel a professional responsibility to help your health system decarbonize and become more resilient to climate change impacts?”* Responses were rated on a Likert scale from 1 (“no responsibility”) to 10 (“very high responsibility”).

The results of the longitudinal survey (Figures 2–8; Supplementary Table S4) are grouped by course objective, demonstrating the course’s impact on participants’ skills and perceptions across four key areas related to climate and health: awareness, communication, preparedness, and professional responsibility.

#### 3.2.1 Climate and health awareness

Compared to the beginning of the course, final survey responses to the question, “To what extent do the impacts of climate change on health affect the work you do in your professional practice?” showed a significant increase in awareness of climate change impacts on professional practice (*p*-value < 0.0001). A pre-course assessment of climate change and health awareness averaged 6.8 on a 10-point scale (SD: 2.6). After completing the course, the average increased to 8.3 (SD: 2.0), reflecting a 22% improvement in participants’ perception of climate change’s impact on their work practices (Figure 2).

#### 3.2.2 Communication

Over the course of the program, participants reported a significant increase in their confidence in communicating the impacts of climate change on health among colleagues and patients (p-value < 0.0001 for both questions). The mean confidence level for communicating with colleagues increased from 6.6 (SD = 2.7) to 9.0 (SD = 1.4), while confidence in communicating with patients improved from 6.4 (SD = 2.8) to 8.8 (SD = 1.6). This represents an approximate 40% improvement in participants’ confidence in effectively communicating the health impacts of climate change (Figures 3, 4).

#### 3.2.3 Preparedness

Over the course of the program, participants reported substantial improvements in their preparedness to support communities in adapting to climate change and to assist health systems in decarbonizing and becoming more resilient to climate change impacts (*p*-value < 0.0001 for both questions). The mean score for preparedness to health communities increased from 6.3 (SD = 2.84) to 8.7 (SD = 1.65), reflecting a mean group change of 2.36 and a 37% improvement (Figure 5). The mean score for preparedness to help health systems decarbonize and enhance resilience rose from 6.1 (SD = 2.93) to 8.3 (SD = 1.86), with a mean group change of 2.18 and a 36% improvement (Figure 6).

#### 3.2.4 Professional responsibility

Longitudinal survey results demonstrated an increased sense of professional responsibility to help communities adapt to the health threats of climate change (*p*-value < 0.0001). The mean score rose from 7.6 (SD = 2.43) to 9.2 (SD = 1.22), reflecting a mean group change of 1.63 and a 21% improvement (Figure 7). Similarly, there was a reported increase in the sense of professional responsibility to help health systems decarbonize and become more resilient to climate change impacts (p-value < 0.0001). The mean score rose from 7.5 (SD = 2.44) to 9.1 (SD = 1.37), reflecting a mean group change of 1.56 and a 21% improvement (SD = 2.44 pre-course and SD = 1.37 post-course; Figure 8).

### 3.3 Final exam

Among the 1,657 participants who completed the exam, 1,481 (89%) achieved a passing score. The mean score was 83.2%, with a standard deviation of 10.4%. Of those who received a certificate of participation, 97% (*n* = 1,607) were from Africa.

## 4 Discussion

Climate change is creating serious challenges for health and health systems, particularly in regions with fragile health infrastructure, like Africa. Health professionals are on the front lines of the climate crisis, yet many barriers prevent health professional engagement and meaningful action to mitigate the root causes of climate change and adapt their health practice to protect patients and communities, especially in vulnerable areas. Rapid knowledge dissemination, capacity building and health professional action is needed to protect patients, communities, and health systems. Following this course, participants from at least 47 African countries and over 35 different health-related fields reported significant improvements in climate and health awareness, communication, preparedness and professional responsibility. These shifts in knowledge, attitude and practice are necessary enablers of a concerted regional response to climate and health impacts. To our knowledge, this is the first free, certificate-based course designed to equip a large number of health professionals in Africa with the knowledge and skills needed to address climate-related health threats effectively.

The results of the longitudinal survey demonstrated a significant shift in participants’ perceptions of how climate change impacts their situational awareness and professional responsibilities, indicating the acquisition of new knowledge and a deeper understanding of climate-health linkages. The analysis of longitudinal questions revealed statistically significant improvement across all domains, with notable increases in average participant scores. Additionally, a consistent reduction in the standard deviation suggests decreased variability in responses, indicating a potential convergence in participants’ knowledge, attitude and practice levels. Overall, these findings highlight a significant improvement in climate change and health awareness (22%), communication (40%), preparedness (36–37%), and professional responsibility (21%) among all participants.

Several specific components of the course likely contributed to the observed improvements in participants’ confidence to address climate and health threats. The inclusion of expert African facilitators provided regionally relevant perspectives and fostered trust and engagement among participants. Multilingual delivery enhanced accessibility and ensured that key concepts were clearly understood across diverse linguistic groups. Additionally, the use of interactive, case-based learning allowed participants to visualize how theoretical knowledge can be applied to practical scenarios. Together, these elements likely strengthened the relevance, cultural resonance, and practical applicability of the training, contributing to the positive learning outcomes reported.

Of those who registered for the Africa Climate and Health Responder course, 66% had never received any training on climate change and health. Although there is no existing data in the literature to confirm if this is a wider trend among African health professionals, we suspect the gaps in education are larger than represented here, given that those who enrolled in this course are likely more attuned to potential impacts and the need for rapid knowledge and skill acquisition. As climate change continues to intensify its impact on the region, concerted efforts are needed to train current and future health professionals from all health sectors. Some of this training must occur at academic institutions, governments and civil society organizations must also create mechanisms to train themselves. Yet, not all institutions are offering this education, leaving gaps in preparedness and capacity (16). According to one of the adaptation indicators on Lancet Countdown regarding the offer of climate change education among global public health institutions, countries with a low human development index (HDI) have the lowest proportion of institutions offering climate change and health education. This disparity suggests that most of the vulnerable nations in Africa may lag in building adaptive health capacities, thereby exacerbating climate change-induced inequities (17).

Climate and Health Responder Courses have been successfully implemented across various regions, including North and Latin America, the Caribbean, Europe, and Southeast Asia (18, 19). In Europe, ASPHER and GCCHE launched a 10-week live-virtual course in early 2024, enrolling approximately 4,600 participants from across Europe and beyond, with over 850 successfully completing the program (18). Similarly, a 2022 Caribbean pilot engaged multidisciplinary health professionals from 37 countries, demonstrating significant improvements in climate-health communication and application through longitudinal survey design (19). While course completion rates have remained consistent with previous iterations, the Africa Climate and Health Responders Course distinguished itself with unprecedented levels of registration and participation, surpassing historical norms—particularly reflecting the high demand for climate-health capacity building in the African context.

The core curriculum of these courses addresses the major evidence-based impacts of climate on health, ensuring a comprehensive understanding of the subject matter and global harmonization of health professional roles and responsibilities (15, 20). However, each regional course is meticulously tailored to incorporate local nuances, reflecting factors such as socioeconomic development, available technology and scientific advancements, and the accessibility of health sector infrastructure. In the African context, cultural aspects must accompany scientific approaches. The course explored how climate change challenges traditional knowledge systems, as increasingly extreme and unpredictable weather patterns threaten to render ancient forecasting methods less effective.

This regional customization ensures that health professionals are equipped with relevant knowledge and practical strategies to address the specific challenges posed by climate change in the continent. By acknowledging and integrating these regional differences, the courses enhance the capacity of health systems worldwide to respond effectively to the evolving climate-health landscape.

The 11-sessions course offered a concise overview of how climate change intensifies weather events, increasing their severity and frequency, and worsening health issues. It highlighted the heightened vulnerability of groups such as women, children, low-income individuals, indigenous communities, those in fragile environments, and people with limited access to essential services like water, sanitation, hygiene (WASH), food, and healthcare. The sessions provided guidance on identifying these at-risk populations and regions, along with case studies on developing adaptive strategies to strengthen health system resilience. By covering these key areas, the course equipped participants with the cutting-edge, practice-ready essential knowledge to develop health systems and practices capable of enduring the challenges posed by climate change.

While education lays the groundwork for progress, Africa confronts significant obstacles in advancing effective climate strategies, including limited data capture and surveillance tools, inadequate infrastructure, insufficient scientific research, and low technological capacity, all of which diminish the continent’s ability to adapt to climate change (9).

Despite these hurdles, Africa is committed to leveraging climate change as a catalyst for transformative development. The African Union’s Agenda 2063 envisions “The Africa We Want,” emphasizing economic transformation, agricultural modernization, and environmental sustainability to position the continent as a future global powerhouse (21). Balancing climate change impacts with economic development presents both challenges and opportunities, highlighting the need to enhance adaptive capacity and resilience across all sectors.

This commitment is evident in the course’s reach, with registrants and survey respondents representing 52 and 47 African countries, respectively. Notably, 31% of course completers were affiliated with government institutions, and another 25% from academia which includes researchers, professors and students. The knowledge gained from the course can now be integrated into curriculum and participants’ practices, enhancing their ability to effectively communicate, prepare for climate-related challenges, and contribute to the adaptation of health systems.

To ensure long-term impact, sustainability must be at the core of these efforts, requiring ongoing investment in education, capacity building, and knowledge-sharing platforms. Programs like the Climate and Health Responders Course play a crucial role in equipping professionals with the necessary skills to drive climate resilience in the health sector. However, scalability faces some barriers including uneven digital infrastructure and internet access across Africa, which can limit equitable participation in virtual trainings. Institutional challenges, such as inconsistent policy support and limited resources, also hinder broader adoption. Sustained policy integration, funding, and institutional commitment are essential not only to expand these initiatives but also to translate individual learning into long-term behavioral and systemic change. Strengthening digital infrastructure and embedding climate-health priorities into national strategies will be crucial for achieving lasting impacts.

### 4.1 Next, steps and future directions

Understanding the impact of this type of training is just as important as tracking the number of people trained. To support this, participants will be invited to complete a (qualitative and quantitative) survey 12 months after completing the course. The survey will explore how the training influenced their engagement in climate-related activities, including adaptation, mitigation, capacity building, policy and advocacy, and research.

Due to the significant vulnerabilities and barriers faced by the African continent in addressing climate and health challenges, a second phase will follow this course. In this phase, 50 selected participants from 25 African countries—representing government, academia, NGOs, and the private sector—will join a Community of Practice to develop and implement a local version of the Climate and Health Responders Course.

This initiative adopts a Train-the-Trainer approach, providing participants with in-depth knowledge of climate change and health, communication strategies, teaching methodologies, and practical tools to develop localized curricula. By the end of this program, these trained responders will begin conducting their own training programs across Africa.

Led by the GCCHE and Project ECHO, and implemented in partnership with the same group of regional collaborators, this initiative aims to make climate change education accessible across the continent. Training health professionals is a critical step in equipping communities and health systems to tackle the climate crisis (22). However, sustaining and scaling this effort requires overcoming persistent barriers and fostering continued support.

To further strengthen the contribution of this initiative to scalable climate-health education in Africa, a comprehensive evaluation framework will be developed to assess the long-term impact of the Train-the-Trainer phase. This will include follow-up surveys to measure skill application, tracking metrics on the reach of localized trainings, and qualitative assessments of policy or community-level outcomes. Trainers will be asked to submit reports at the end of the program and 1 year after implementation. These findings will help identify barriers to program continuity, inform next steps, and provide evidence of sustained impact on health systems and resilience.

To enhance climate and health education among African health professionals, the following recommendations are proposed:

1. Policymakers should develop and enforce policies that promote climate change education and preparedness within the health sector.
2. Governments and private sectors should offer specialized training to current health professionals, enhancing their capacity to manage climate-related health issues.
3. Academic institutions should embed climate change and health topics into their programs to equip future health professionals from all backgrounds with essential knowledge.
4. Government, academic institutions, private sector and NGOs must design preparedness training that reflects local challenges, incorporating region-specific case studies to ensure relevance and effectiveness.
5. Health professionals should pursue ongoing education in climate and health to deepen their expertise and maintain readiness for climate-related challenges.
6. Health professionals empowered with knowledge must work alongside governments in educating communities on climate adaptation measures.

## 5 Limitations

The study relied heavily on self-reported survey data to measure changes in knowledge, confidence, preparedness, and professional responsibility. This approach is subject to social desirability bias and may not accurately reflect actual skill or knowledge acquisition. The study relies on self-selected participants who registered for the course through outreach conducted via professional networks with a strong interest in climate and health. This may introduce selection bias, as those already interested in climate and health may be more likely to participate and show positive changes. As a result, the findings may not be generalizable to the broader population of health professionals, particularly those with less awareness, exposure, or perceived relevance of climate-health intersections. Additionally, as the course was conducted online, participants with limited access to reliable internet or appropriate devices may have faced difficulties fully engaging, affecting their learning experience. While the course included region-specific case studies, some areas of Africa may not have been equally represented, limiting the applicability of certain lessons to all participants. Additionally, while the course aimed to feature experts from diverse countries, the sessions may not fully capture the vast diversity of Africa’s health systems and the significant regional variations within the continent. Finally, we acknowledge that course completers may differ from non-completers, introducing potential bias. Thus our findings should be interpreted as a reflection of the experiences of those who completed the course.

## 6 Conclusion

Health professionals play a vital role in addressing climate change through mitigation and adaptation efforts. Their expertise and influence as health messengers remain underutilized in society’s response to this crisis. Regionally tailored, live-virtual, evidence-based courses have shown great promise in transforming health professionals’ approaches to tackling climate-related health challenges. The Africa Climate and Health Responders Course is a testament to this, equipping participants with the knowledge and skills needed to improve awareness, communication, and preparedness.

For Africa to effectively adapt to climate change and build resilient health systems, sustainable efforts involving multiple stakeholders are essential. Advancing climate and health education requires integrating climate topics into health curricula, providing specialized training, and engaging communities. These initiatives not only strengthen health systems but also position Africa as a leader in climate adaptation and resilience, paving the way for a healthier and more sustainable future. Continued funding for the GCCHE Climate and Health Responders Course is essential to equipping health professionals with the knowledge and skills needed to address the growing health impacts of climate change. Sustained investment ensures that this critical training reaches frontline responders, strengthens health systems, and fosters global resilience in the face of climate-related health threats.

## Data Availability

The original contributions presented in the study are included in the article/Supplementary material, further inquiries can be directed to the corresponding author.
