# Supplementary material for: Improving health professionals’ capacity to respond to the climate crisis in Africa: outcomes of the Africa climate and health responder course
Source: Front Public Health. 2025 Oct 15;13:1617723. doi: 10.3389/fpubh.2025.1617723 (PMC12568577; doi:10.3389/fpubh.2025.1617723)
Supplement: Supplementary file 1 [file Data_Sheet_1.docx]

**Appendix 1 - Curriculum**

**S1 Appendix - Didactic Curriculum: African Climate and Health Responder Course**

1) Climate Change for the Health Professional

This session provided an in-depth exploration of the intersection between climate change and human health, emphasizing the crucial role of health professionals in engaging in climate mitigation, adaptation, and resilience strategies, and current policy priorities in the African context. Through case studies and discussions, the session explored how climate change disproportionately impacts socioeconomically, racially, and ethnically marginalized communities both locally and globally, leading to disparate health outcomes and the urgency of trans-sectoral solutions in addressing these challenges.

2) Extreme Weather Hazards: Tropical Cyclones and Flooding

In this session, we explored the increasing risks of extreme weather events, such as tropical cyclones, inland and coastal flooding, driven by climate change in Africa. Participants gained insights into the short-term and long-term health threats posed to patients and communities impacted by these events, and learned about actionable steps that health professionals could take within their roles to mitigate these risks. We delved into the various biological, social, economic, and structural factors that contribute to the vulnerability of individuals and populations to health impacts from extreme weather hazards. Additionally, we defined the critical roles of disaster risk reduction, public health communication, early warning systems, and regional cooperation in preventing the health impacts of extreme weather events and minimizing migration.

3) Water-and Food Related Illness

This session provided an in-depth examination of how climate change impacts the hydrosphere, including the occurrence of droughts and heavy precipitation, and the consequent implications for water availability, quality, and supply. Participants explored the biologic, social, economic, and structural factors that contribute to the vulnerability and affect the incidence and prevalence of waterborne pathogens. Through case studies and discussions, participants gained insights into the complex relationship between climate change and waterborne diseases, as well as strategies for prevention and mitigation. Through interactive discussions, case studies, and practical examples, this session aimed to equip health professionals with the knowledge and skills necessary to recognize, address, and prevent the health impacts of climate-related impacts to water and foodborne illness in African communities.

4) Heat Related Illness and Mortality

This session focused on current and projected changes to temperature and the increasing risks of extreme heat events in Africa. We delved into the concept of "heat-sensitive" health conditions and explored how heat impacts the pathophysiology of various diseases, including cardiovascular, renal, respiratory, neurologic, mental health, and maternal conditions while identifying medical diagnoses and other health determinants that render patients more vulnerable to heat related illness. We explored the role of heat early warning systems and other preventive measures in averting negative health outcomes, emphasizing the importance of proactive strategies in mitigating heat-related health risks. Through interactive discussions, case studies, and practical examples, this session aimed to equip health professionals with the knowledge and skills necessary to recognize, address, and prevent the health impacts of extreme heat events in African communities.

5) Degraded Air Quality

This session provided a comprehensive exploration of the pathways through which climate change affects air quality in Africa, including changes to ozone, PM2.5, wildfires, desert dust, and other ambient respiratory irritants. Participants learned how these pollutants impact populations vulnerable to degraded air quality, considering factors such as socioeconomic status, geographic location, and pre-existing health conditions. Participants will gain insights into public health measures to protect vulnerable populations and explore strategies for community-based interventions. Furthermore, the session delved into the near-term health co-benefits of climate mitigation efforts, including improved air quality, at the individual, local, and global scales. Through interactive discussions, case studies, and practical exercises, participants gained insights into examples of successful mitigation strategies, highlighting the potential for positive health outcomes associated with climate action.

6) Vector-borne and Zoonotic Diseases

This session provided a comprehensive exploration of the environmental processes changing as a result of climate change and their impact on the prevalence, incidence, and distribution of vector-borne and zoonotic diseases including Malaria, Dengue fever, Rift Valley fever, Schistosomiasis, Chikungunya, and Zika. Through case studies we explored actionable steps that health professionals can take to identify and protect patients vulnerable to these diseases through targeted interventions, including early warning systems, and community-based, interdisciplinary approaches to disease prevention and control in a changing climate.

7) Climate Extremes, Agriculture and Food Security

This session focused on the complex interplay between extremes of temperature and precipitation and their impacts on food security and malnutrition in the African continent. Participants gained insights into how climate change-induced variations in temperature and precipitation patterns affect agricultural productivity and exacerbate food insecurity and malnutrition. Moreover, participants explored mechanisms for forecasting food insecurity using climate models, understanding the role of predictive tools in identifying vulnerable regions and populations and informing proactive interventions. Through case studies and discussions, participants explored examples of strategies to improve food security in a changing climate and learned about innovative approaches such as drought-resistant crop varieties, sustainable agricultural practices, and community-based adaptation initiatives.

8) Climate Change, Mental Health and Forced Migration

This session delved into the profound impact of climate change on the incidence and prevalence of stress disorders, depression, anxiety, suicide, domestic abuse, violence, aggression, and substance abuse as a consequence of fast and slow onset climate-related disasters. Through case studies and discussions, participants gained insights into the complex interplay between environmental stressors and mental health outcomes. We also examined the specific mental health challenges faced by "climate refugees" or individuals displaced from their homes or livelihoods due to climate-related environmental changes such as drought, sea-level rise, wildfires, or hurricanes, with a focus on recent examples from Africa. Through interactive discussions and case studies, we explored the role of resilience-building interventions, community-based support systems, and mental health services in promoting psychological well-being in the face of environmental challenges.

9) National Adaptation Planning and Vulnerability Assessments

This session focused on national adaptation planning and vulnerability assessments as essential tools for understanding and addressing the impacts of climate change on health systems and communities and creating robust policy action. Participants explored how vulnerability assessments are utilized to understand individual, community, and health system vulnerabilities, in order to inform comprehensive climate and health planning and response. Additionally, participants learned about the importance of collaboration and information sharing across departments, ministries, and agencies to support integrated surveillance and response efforts to guide effective climate adaptation and health promotion.

10) Health Service Delivery Sustainability and Adaptation

This session focused on applying the concepts of sustainability and adaptation to the healthcare sector and explored practical examples of how healthcare systems can embody both principles, thereby improving health service delivery in a changing climate. Through case studies and discussions, participants gained insights into how healthcare facilities can become more resilient in the face of increasingly severe and frequent climate-related weather extremes through addressing workforce surge needs, ensuring the continuity of critical infrastructure for healthcare and emergency services, and understanding the roles and interactions between various agencies involved in emergency care. Furthermore, the session highlighted the role of health professionals in partnering with healthcare institutions, professional organizations, and advocacy groups to reduce the healthcare sector's greenhouse gas footprint and foster partnerships for a more sustainable and resilient healthcare system.

11) Climate and Health Research and Funding in Africa

This session featured an engaging panel discussion with representatives from leading funding institutions dedicated to supporting climate and health research in Africa. The session aimed to explore the current landscape of funding opportunities, discussed the challenges and opportunities in securing research grants, and highlighted successful projects that have made significant impacts on climate and health outcomes in Africa. Panelists shared insights on the criteria and priorities for funding, strategies for building successful grant proposals, and the future direction of funding in this critical area. Attendees gained a deeper understanding of the available resources and how to navigate the complex funding ecosystem to support innovative research and interventions in climate and health.
