# Supplementary material for: Improving health professionals’ capacity to respond to the climate crisis in Africa: outcomes of the Africa climate and health responder course
Source: Front Public Health. 2025 Oct 15;13:1617723. doi: 10.3389/fpubh.2025.1617723 (PMC12568577; doi:10.3389/fpubh.2025.1617723)
Supplement: Supplementary file 2 [file Data_Sheet_2.docx]

**Appendix 2 - Registration and Longitudinal Questionnaire**

**Registration Questionnaire**

1. First Name
2. Last Name
3. Email
4. Country/Region
5. Organization
6. Role at Organization
7. Place of Employment
8. Background
9. Are you a student?
10. Have you received any prior training in climate and health?
11. Which language do you prefer?

**Longitudinal Questionnaire**

LG1. To what extent do the impacts of climate change on health affect the work you do in your professional practice?

1. "Not relevant - Climate change does not impact my professional practice"
2. "To a large extent - Climate change impacts all facets of my professional practice"

LG2. How confident do you feel communicating with WORK COLLEAGUES about the health impacts of climate change?

1. "Not Confident"
2. “Very Confident"

LG3. IF APPLICABLE: How confident do you feel communicating with PATIENTS about the health impacts of climate change?

1. "Not Confident"
2. “Very Confident"

LG4. To what degree do you feel prepared to: Help your community adapt to the health threats of climate change?

1. "Unprepared"
2. "Prepared"

LG5. To what degree do you feel prepared to: Help your health system decarbonize and become more resilient to the impacts of climate change?

1. "Unprepared"
2. "Prepared"

LG6. To what degree do you feel a sense of professional responsibility to: Help your community adapt to the health threats of climate change?

1. "No Responsibility"
2. "Very High Responsibility"

LG7. To what degree do you feel a sense of professional responsibility to: Help your health system decarbonize and become more resilient to the impacts of climate change?

1. "No Responsibility"
2. "Very High Responsibility"
